# Relationship between patient safety culture and patient experience in hospital settings: a scoping review

**DOI:** 10.1186/s12913-024-11329-w

**Published:** 2024-08-07

**Authors:** Adel Alabdaly, Reece Hinchcliff, Deborah Debono, Su-Yin Hor

**Affiliations:** 1 Faculty of Health, University of Technology Sydney, Sydney, NSW Australia; 2 College of Nursing, Imam Abdulrahman Bin Faisal University, Dammam, Eastern Province Saudi Arabia; 3 School of Applied Psychology, Griffith Health Group, Griffith University, Brisbane, QLD Australia; 4 School of Public Health and Social Work, Faculty of Health, Queensland University of Technology, Brisbane, QLD Australia; 5 School of Public Health, University of Technology Sydney, Sydney, NSW Australia

**Keywords:** Safety culture, Safety climate, Patient experience, Patient satisfaction, Customer satisfaction, Healthcare quality, Health services, Quality indicators, Patient safety, Hospital

## Abstract

**Background:**

Measures of patient safety culture and patient experience are both commonly utilised to evaluate the quality of healthcare services, including hospitals, but the relationship between these two domains remains uncertain. In this study, we aimed to explore and synthesise published literature regarding the relationships between these topics in hospital settings.

**Methods:**

This study was performed using the five stages of Arksey and O’Malley’s Framework, refined by the Joanna Briggs Institute. Searches were conducted in the CINAHL, Cochrane Library, ProQuest, MEDLINE, PsycINFO, SciELO and Scopus databases. Further online search on the websites of pertinent organisations in Australia and globally was conducted. Data were extracted against predetermined criteria.

**Results:**

4512 studies were initially identified; 15 studies met the inclusion criteria. Several positive statistical relationships between patient safety culture and patient experience domains were identified. Communication and teamwork were the most influential factors in the relationship between patient safety culture and patient experience. Managers and clinicians had a positive view of safety and a positive relationship with patient experience, but this was not the case when managers alone held such views. Qualitative methods offered further insights into patient safety culture from patients’ and families’ perspectives.

**Conclusion:**

The findings indicate that the patient can recognise safety-related issues that the hospital team may miss. However, studies mostly measured staff perspectives on patient safety culture and did not always include patient experiences of patient safety culture. Further, the relationship between patient safety culture and patient experience is generally identified as a statistical relationship, using quantitative methods. Further research assessing patient safety culture alongside patient experience is essential for providing a more comprehensive picture of safety. This will help to uncover issues and other factors that may have an indirect effect on patient safety culture and patient experience.

## Introduction

Patient safety is a pressing challenge for health systems, globally. The importance of promoting and sustaining a robust safety culture is widely recognised [1]. The importance of the patient’s role in supporting patient safety is also increasingly recognised [2]. Despite the prominence of the concepts of patient safety culture and patient experience in academia and industry, the relationship between them remains underexplored and diffuse.

The concept of patient safety culture was defined as a collective of beliefs, attitudes, values, and norms that influence behaviours and attitudes, concerning patient safety [3]. Patient perspectives are often neglected when measuring safety culture [4]. Patient experience has been defined as patients’ perspectives of services, recognising that patients are the most valuable sources of information about their experiences [5].

It is essential to put the patient at the centre of healthcare services [6], and to do this requires nurturing caring cultures through the assurance that health professionals feel esteemed, involved and supported [7]. Patients pay attention to staff performance and other issues and can identify safety problems that hospital staff may miss, such as problems entering and exiting the healthcare system, systemic (multiple and distributed) problems that are cumulative, and errors of omission, especially the failure to attend to patients’ concerns [2, 8–10]. A cultural change from the conventional approach that considered patients as care recipients, to seeing patients as partners in their care, is essential to provide patient-centred care that is informed by patient experience.

There has been considerable knowledge gained about patient safety, but it persists as a worldwide challenge in healthcare [11], with serious incidents and iatrogenic harm continuing to occur across health care settings, including within hospital settings. There has been a focus on reducing iatrogenic harm by enhancing safety culture in hospitals.

Understanding patient safety from the staff perspective alone is not enough. It is essential to also understand what factors might link safety culture and patient experience, as concepts often measured separately, but both important indicators of safety and quality. In examining this link, we hope to better understand what facets of care might contribute to both safety culture, as experienced by staff, and the safety and quality of care, as experienced by patients. The aim of this review is to explore and synthesise existing research literature to find out what is known regarding the relationship between patient safety culture and patient experience (of safety and quality) in hospital settings. We sought to achieve this aim through the following objectives: (a) to identify how these concepts have been defined or described in the literature; (b) to identify how these concepts are measured; and (c) to identify the links between the concepts.

## Methods

This study followed a published protocol [12]. The methodology of this scoping review was developed using the Arksey and O’Malley [13] framework for a scoping review (Arksey & O’Malley, 2005), refined by the Joanna Briggs Institute [14]. The Preferred Reporting Items for Systematic Reviews and Meta-Analyses extension for Scoping Reviews (PRISMAScR) [15] guidelines were followed. The study does not critically appraise the included papers’ quality and risk of bias. The aim in our scoping review is not to evaluate the quality of the evidence found, but rather to explore what research has been done in this field, and what approaches were undertaken.

The processes of searching, applying inclusion and exclusion criteria, screening, data extraction, and reporting of the findings followed a published protocol for this study [12]. The search terms and strategies appear in the protocol, and searches were completed on 18 June 2022.

### The inclusion and exclusion criteria

This review followed the Population, Concept and Context (PCC) framework for the inclusion criteria recommended by the Joanna Briggs Institute for scoping reviews [14]. In addition to the PCC criteria noted in Table 1, included studies must have been conducted in the hospital context and reported in English or Arabic languages.

**Table 1:** The PCC framework used in the scoping review

PCC	Inclusion Criteria
**Population**	• Healthcare providers in hospital contexts, including management, clinical and non-clinical staff. • Patients who have received healthcare services in hospital settings, irrespective of demographic characteristics.
**Concept**	Any article that focuses on patient safety culture, safety climate or organisational culture, in addition to patient experience or patient satisfaction.
**Context**	Hospital setting.

### Search

We searched journals from seven electronic databases relevant to the scope of the study (CINAHL, Cochrane Library, ProQuest, MEDLINE, PsycINFO, SciELO and Scopus); web search engine Google Scholar (first 30 results); and four organisations in Australia and globally: the Agency for Healthcare Research and Quality (AHRQ), the Australian Commission for Safety and Quality in Healthcare (ACSQHC), the Agency for Clinical Innovation (ACI), and National Institutes of Health (NIH). We supplemented these searches with hand-searching the reference lists of the final included papers for additional studies of relevance.

### Study selection

As indicated in the protocol for this study [12], retrieved papers were screened and selected in two phases. In the first phase, one reviewer (AA) evaluated all titles and abstracts to determine whether each paper met the eligibility criteria, including categorising screened studies as ‘included’, ‘excluded’ or ‘not sure’. All papers screened as ‘included’ and ‘not sure’ in the first phase were considered for full-text review by the reviewer (AA). In the second phase, three reviewers (RH, DD, SH) screened ten per cent of titles and abstracts of studies screened as ‘included’, ‘excluded’ or ‘not sure’ against selection criteria. All authors (AA, RH, DD, SH) independently reviewed the full text of the included studies. The authors discussed the included papers in a meeting and reached a consensus on the included papers, with no disagreement between the authors.

### Charting the data

One reviewer (AA) extracted relevant data from the included studies to address the scoping review question using the template provided in the published protocol [12]. Three reviewers (RH, DD and SH) verified the accuracy of the data extraction exercise. The data extracted included the following:

- Author/s.
- Country.
- Aims/objective(s).
- Methodology/methods.
- Inclusion/exclusion criteria (e.g., PCC).
- Types of intervention (if applicable).
- Measurement of outcomes (if applicable).
- Key results that relate to the review question.

### Reporting the findings

Other concepts related to patient safety culture and patient experience, such as safety climate and patient satisfaction, were used in literature that measured safety culture or patient experience. The nuances of these terms were illustrated in the published protocol. The decision was taken to incorporate findings about safety climate alongside those about patient safety culture, and to incorporate findings about both patient satisfaction and patient experience. We noticed that the ‘patient experience’ and ‘patient satisfaction’ terms are often used interchangeably. For example, a study conducted by Mazurenko et al. [16] used the term ‘patient satisfaction’ in the paper title but measured patient satisfaction using the HCAHPS tool, which is a well-known tool for measuring ‘patient experience’. In fact, the terms, as operationalised in the instruments, overlap more than they should.

According to Bull [17], ‘patient satisfaction’ involves an evaluation and hence is subjective, suggesting that ‘patient experience’ is the more objective measure. However, considering the questions in the HCAHPS tool (commonly used for measuring ‘patient experience’ as mentioned above), we see that several questions involve an element of subjectivity and evaluation from the patient’s perspective. For instance, questions like: “During this hospital stay, how often did nurses treat you with courtesy and respect?” or “How often did you get help in getting to the bathroom or in using a bedpan as soon as you wanted?”. The point made by Bull [17] reflects a tension between the recognised importance of finding out what care is like, from patients’ perspectives (which is subjective and evaluative), and the desire for objective measurements of care delivery for the purposes of comparison and evaluation of health services [18]. Due to these concepts being so intertwined in how they are understood and measured, and not wanting to limit the understanding of the patient experience only to objective measures devoid of patients’ subjective judgements, papers on patient satisfaction from the review were included, based on the inclusion criteria.

The study sought to review a wide range of literature in relation to the study aim and inclusion criteria. Rather than being a systematic review or meta-analysis, the study aims to offer the reader an overview of the research carried out regarding the relationship between safety culture and patient experience. The characteristics and findings of the included papers were analysed initially by (AA), performing a content analysis, using a framework of categories aligned with the research questions. Within these categories, study features and findings were discussed among all the authors (AA, RH, DD, SH), and descriptively summarised. All authors agreed upon the findings and categories. This descriptive content analysis was found to be sufficient to address the study objectives. Thus, deviating from the published protocol [12], no further thematic analysis was conducted. The results are presented according to the categories as follows:

- Conceptualisations of patient safety culture and patient experience.
- Measurement of patient safety culture and patient experience.
- Relationship between patient safety culture and patient experience.

## Results

**Fig. 1:**
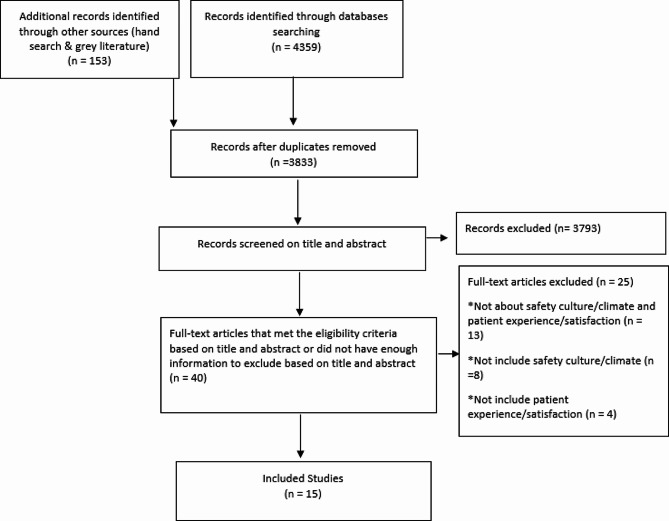
PRISMA flowchart of search process and results

As depicted in Fig. 1, the initial search yielded 4512 articles. After removing duplicates, 3833 articles remained, and 3793 were excluded at the first stage of screening (title and abstract). Following full-text screening, 15 articles remained that met the inclusion criteria. The included studies were conducted in different countries, including Australia (one study) [19], Canada (two studies) [8, 20], Germany (one study) [4], Indonesia (one study) [21], Iran (one study) [22], Israel (two studies) [10, 23], Nigeria (one study) [24], United Kingdom (one study) [2] and United States (five studies) [16, 25–28]. A summary of the characteristics of the included studies is presented in Table 2.

**Table 2:** Characteristics of included studies

Author(s)	Year	Country	Methods	Concepts noted in the included paper
Lawton R, O’Hara JK, Sheard L, Reynolds C, Cocks K, Armitage G, et al. [2]	2015	UK	Quantitative surveys	Safety culture, patient experience, patient perceptions of safety
Monaca C, Bestmann B, Kattein M, Langner D, Müller H, Manser T. [4]	2020	Germany	Quantitative surveys	Safety culture, safety climate, patient satisfaction, patient experience of safety culture
Bishop AC, Cregan BR. [8]	2015	Canada	Qualitative interviews	Safety culture, patients experience, family experience, patient satisfaction
Kagan I, Porat N, Barnoy S. [10]	2019	Israel	Quantitative surveys	Safety culture, organizational culture, patient satisfaction, patient experience
Mazurenko O, Richter J, Kazley AS, Ford E. [16]	2019	US	Quantitative surveys	Safety culture, safety climate, organizational culture/culture, patient satisfaction, patient experience
Do VQ, Mitchell R, Clay-Williams R, Taylor N, Ting HP, Arnolda G, Braithwaite J. [19]	2021	Australia	Quantitative surveys	Safety culture, safety climate, patient experiences, patient perceptions of safety
Dodek PM, Wong H, Heyland DK, Cook DJ, Rocker GM, Kutsogiannis DJ, et al. [20]	2012	Canada	Quantitative surveys	Safety culture, organizational culture, family satisfaction, consumer satisfaction, patient experience
Sembodo T, Hadi C, Purnomo W. [21]	2019	Indonesia	Quantitative surveys	Safety culture, organizational culture, patient satisfaction, customer satisfaction
Afshar PJ, Karbasi BJ, Moghadam MN. [22]	2021	Iran	Quantitative surveys	Safety culture, patient satisfaction
Burlakov N, Rozani V, Bluvstein I, Kagan I. [23]	2021	Israel	Quantitative surveys	Safety climate, patient satisfaction, family satisfaction, patient experience
Okafor CH, Ugwu AC, Okon IE. [24]	2018	Nigeria	Quantitative surveys	Safety culture, patient satisfaction, patient experience
Abrahamson K, Hass Z, Morgan K, Fulton B, Ramanujam R. [25]	2016	US	Quantitative surveys	Safety culture, organizational culture, patient experience, patient satisfaction
Lyu H, Wick EC, Housman M, Freischlag JA, Makary MA. [26]	2013	US	Quantitative surveys	Safety culture, safety climate, safety attitudes, patient satisfaction, patient’s experience
Smith SA, Yount N, Sorra J. [27]	2017	US	Quantitative surveys	Safety culture, organizational climate, safety climate, patient experience
Sorra J, Khanna K, Dyer N, Mardon R, Famolaro T. [28]	2012	US	Quantitative surveys	Safety culture, patient experience, patient satisfaction

### Conceptualisations of patient safety culture and patient experience

#### Patient safety culture

In the studies reviewed, patient safety culture was commonly conceptualised as relating to the attitudes, beliefs, perceptions, norms and values that workers share about safety [8, 10, 24, 27]. These shared characteristics shape healthcare professionals’ understandings of what is essential in a healthcare institution, how they should act, what attitudes or actions are acceptable, and what approaches are rewarded or punished concerning patient safety [8, 10, 27]. Patient safety culture has been identified within the included studies as being central to the behaviour of the individuals, and influences staff proficiency, attitudes and behaviours concerning their safety performance [8, 10, 27].

The reviewed literature also identified patient safety culture as one element of a broader organisational culture, related to preventing and detecting shortfalls in patient safety, and managing patient safety in healthcare settings [16, 20, 21]. The concept of ‘safety climate’ was also prevalent in the literature, and was often used in studies that also described ‘safety culture’ [10, 16, 19, 26, 27] without distinguishing between the two concepts.

#### Patient experience

From our review of the studies, the concept of patient satisfaction was more commonly used than patient experience, and defined as a subjective assessment of the ways those receiving healthcare react to particular relevant elements of treatment, including the process, environment, and outcomes, and this was quantified as representing the degree to which patients believe that their requirements and aspirations were fulfilled by their experiences [24, 26]. Although the research that examined patient experience, did not offer specific definitions of the concept, patient experience was conceptualised as a resource for understanding patients’ perceptions, which helps promote the quality and safety of healthcare services [2, 8, 25, 27, 28].

The reviewed research frequently refered to the concept of patient satisfaction and ways of measuring it, regarding patient satisfaction as indicative of the effectiveness of organisational performance with regard to patient safety [2, 8, 25–27]. Review of the included studies identified another related concept, customer satisfaction, which is defined as how the individual feels when making a comparison between what they expected and how they regarded what they received; this is regarded as a high-performance target for the delivery of public services [21]. The variation in the concepts also reflected variation in the measurement tools currently used.

### Measuring patient safety culture and patient experience

In the research reviewed, patient safety culture was most commonly measured by the deployment of questionnaires. Included studies also presented assessments of the validity of deployed instruments. The most common patient safety culture tool used in the reviewed studies was the Hospital Survey on Patient Safety Culture (HSOPS) [2, 16, 20, 22, 24, 25, 27, 28]. The next most common tool used was the Safety Attitudes Questionnaire (SAQ) [19, 26]. The SAQ was also combined with the Leadership Effectiveness Survey (LES) to construct a new tool named the Safety Culture and Leadership Questionnaire to assess clinician perceptions of safety, teamwork and leadership [19].

The HSOPS tool developed by the Agency of Healthcare Research and Quality was employed in included studies to assess clinician and staff perceptions of the culture of safety at the hospital’s macro level [16, 22, 27, 28]. HSOPS is also used in individual departments within a hospital [2, 20, 24, 25], and regarded as a reliable and valid tool. The SAQ is another reliable and valid tool employed for the evaluation of patient safety culture [26]. The safety culture domains in HSOPS and SAQ tools are different but overlapping (Table 3).

**Table 3:** Patient safety culture dimensions in the SAQ and the HSOPS

HSOPS	SAQ
1. Communication openness 2. Feedback & communication about error 3. Frequency of events reported 4. Handoffs & transitions of patient information 5. Management support for patient safety 6. Non-punitive response to error 7. Organisational learning and continuous improvement 8. Overall perceptions of patient safety 9. Staffing 10. Supervisor/manager expectations and actions promoting safety 11. Teamwork across units 12. Teamwork within units	1. Teamwork climate 2. Job satisfaction 3. Perceptions of management 4. Safety climate 5. Working conditions 6. Stress recognition

The use of HSOPS and SAQ tools reflected the overlap in use of the concepts of safety culture and safety climate. For example, HSOPS includes more dimensions of patient safety culture than the SAQ, and both tools were employed to measure ‘patient safety culture’ [2, 16, 20, 21, 24–28], although the HSOPS was also employed for the measurement of ‘safety climate’ [16]. In addition, the SAQ includes two dimensions referring to climate: teamwork climate and safety climate [29]. Importantly however, both the HSOPS and SAQ offer a quantitative measure of patient safety culture from the point of view of staff alone [2, 16, 20, 24–28].

Patient-reported measures of safety were limited and mentioned more frequently in more recent literature. The Patient Measure of Safety (PMOS), Patients’ Perceptions of Safety Culture (PaPSC) and narratives were used in the research reviewed to identify safety concerns from the patient’s perspective and provide data regarding safety matters, including patient safety culture [2, 4, 8, 19]. Lawton et al. [2] noted that the PMOS has undergone considerable testing and is generally recognised as having both validity and reliability; it is also popular with patients and allows researchers to assess how patients perceive the ways in which organisational elements influence patient safety within a hospital by collecting patient feedback about contributing factors to safety incidents [2].

With regard to measuring patient experience, the Hospital Consumer Assessment of Healthcare Providers and Systems (HCAHPS) was the most frequently used tool in studies reviewed, and is regarded as a valid and reliable instrument for measuring the ways in which patients perceive their interactions with the hospital, and can be used by government as a tool for assessing hospital funding [16, 25, 26, 28]. HCAHPS (also referred to as Hospital CAHPS) asks the patient to report on their recent experiences with inpatient care [16, 25, 26, 28]. The HCAHPS tool measures the following domains: nurse communication, doctor communication, pain management, staff responsiveness, hospital environment, communication about medicine, discharge information, and overall patient perception [16, 25, 26, 28]. Similarly to the overlapping concepts described with the safety culture surveys earlier, the HCAHPS has been employed for the measurement of both patient satisfaction [16, 26] and patient experience [25, 28]. Other feedback tools such as the Patient Satisfaction Questionnaire Short Form (PSQ) [24], the Friends and Family Test (FFT) [2] and Family Satisfaction in the Intensive Care Unit questionnaire (FS-ICU-24) [20] were used for measuring patient feedback and perception of care in our reviewed studies.

Finally, only one study in our review used a qualitative method to examine patient experience; drawing on pre-recorded video narratives published on the Canadian Patient Safety Institute website [2].

### Relationship between patient safety culture and patient experience

In the research reviewed, the relationship between patient safety culture and patient experience was generally identified and presented as a statistical correlation [2, 16, 24–28]. Positive correlations were found between some domains of patient safety culture and patient experience (Table 4) [2, 8, 20, 21, 23, 25, 28]. The teamwork and communication domains seem to be central to positive correlations between patient safety culture and patient experience [8, 16, 25–27]. Other studies reviewed demonstrated no correlation between patient safety culture and patient experience overall scores [2, 24, 26].

Staff responsibilities, including direct contact with patients, may affect the relationship between patient safety culture and patient experience. For instance, no significant correlation was found between patient satisfaction and safety climate when management alone had a highly positive view of the safety climate [16]. However, when management and clinicians both had a positive view of the safety climate, there was a positive correlation. The FFT tool that measured patient experience was correlated with the ways patients perceived safety issues but was not correlated with either the staff safety culture or publicly available safety data [2]. From the sole qualitative study, we find that structuring safety and quality based on teamwork among healthcare professionals, patients, and family members is a more effective approach than relying on the individual healthcare practitioner alone [8]. Also, patients’ and families’ involvement is essential for creating a trusting relationship, which helps create an inviting environment that facilitates and encourages open communication and coordination among staff and patients [8]. Finally, conversation between staff, patients and families is crucial to capture different views of safety and better understand safety culture, particularly from the patient’s perspective.

The research under review also frequently examined how patient safety culture and patient experience, either individually or in combination, were related to other quality measures such as hospital performance, however this is outside of the scope of our review.

**Table 4:** Associated aspects of safety culture and patient experience

Factors that relate to staff	Factors that relate to patient
1. Communication openness 2. Feedback & communication about error 3. Frequency of events reported 4. Handoffs & transitions of patient information 5. Organisational learning and continuous improvement 6. Staffing 7. Teamwork across units 8. Teamwork within units 9. Overall perceptions of patient safety	1. Responsiveness of hospital staff 2. Patient and family engagement & empowerment 3. Discharge information 4. Communication about medications 5. Nurse communication 6. Doctor communication 7. Likelihood to recommend hospital. 8. Hospital environment 9. Transition of care 10. Overall experience

## Discussion

### Patient safety culture and patient experience overlapped with other concepts

The concepts “safety culture” and “safety climate” were used interchangeably in the reviewed literature, which reflects their overlap in the broader literature, although these concepts are also sometimes differentiated. Patient safety culture tends to refer more broadly to the complex set of shared perceptions about safety that form over time in an organisation, while safety climate is considered ‘a snapshot’ of these shared perceptions, that can be measured at a specific time point using survey studies [29, 30].

In the reviewed studies, the use of the terms patient experience and patient satisfaction also significantly overlapped. The two terms are recognised quality indicators for assessing healthcare quality, and while both concepts are related, they have also been differentiated [31]. Although the reviewed studies did not offer specific definitions, patient experience has been described elsewhere as patient “perceptions of phenomena for which they are the best or only sources of information, such as personal comfort or effectiveness of discharge planning” [5 p1]. While patient experience is viewed as the sum of all interactions that influence patient perceptions over the entire experience [32], as noted earlier, patient satisfaction is more about whether patients’ expectations are met [33]. In this regard, patient satisfaction is viewed as evaluating the patient experience of health services. Therefore, patients’ perception of what they actually experienced in healthcare organisations (patient experience) has an influential impact on how they evaluate healthcare services (patient satisfaction).

### Measuring the relationship between patient safety culture and patient experience

The relationship identified between patient safety culture and patient experience in the reviewed literature is mostly measured by quantitative approaches/surveys, and thus little is known about causality or the underlying reasons (or mechanisms) for any relationship identified between these concepts. The availability, validity and reliability of the surveys such as HSOPS and HCAHPS may facilitate and encourage the use of questionnaires in busy working environments such as hospitals. However, the significant differences and variations in methodologies/tools (including dimensions captured by the instruments) employed to measure safety culture and patient experience, makes it difficult to compare the different items of research, and results in variations in the findings.

### Patient involvement in the measurement of patient safety culture

Our review findings support research arguing that patients can provide useful feedback on safety [34]. Patient voice is increasingly included in other aspects of patient safety, but we need to include it more in the measurement of safety culture. In fact, some measures of patient experience pay attention to safety, for instance, in terms of physical comfort and a safe environment, which are also domains of patient safety culture. It was recognised in the included studies that instruments for assessing patient perceptions could be adapted to incorporate questions regarding patient safety, such as PMOS and PaPSC. This would enable patient perceptions and experience of safety to be assessed and the findings employed to effect enhancements in safety culture.

The PMOS and PaPSC scales were developed specifically to capture patients’ feedback on the safety of their care. The PMOS is based on the Yorkshire Contributory Factors Framework (YCFF) to capture patient feedback regarding the contributing factors to patient safety incidents [35]. However, the YCFF was developed based on input from healthcare professionals alone [36]. Likewise, the PaPSC scale was also initially developed based on staff perceptions. Although these scales are administered to patients, they may not fully reflect the patients’ perceptions of safety culture, if patients identify other aspects. In addition, the PMOS data was collected from one hospital in northern England; as such, the outcomes of the survey are not reflective of the perceptions of the general global population.

Another measurement approach for capturing patient perceptions of safety culture is to consider patients’ and families’ pre-recorded narratives as a qualitative assessment method [8]. This approach was limited in terms of inability to ask questions or follow-up with the participants, and the analysis was based on a revised or edited perspective that could carry certain biases. However, this study demonstrated the value of patient narratives and interviews in understanding the interrelationships between different aspects of patient safety culture. In contrast to surveys, qualitative interviews aim to understand participants’ attitudes, behaviours, experiences and perceptions. Qualitative research methods are common in healthcare research, but are largely missing in research into the association between safety culture and patient perceptions of safety culture.

No consensus exists as to the best method to be employed for the measurement of the concepts in question. Different measurements have been employed for each concept for various purposes, resulting in variations in data sources, and variations in results. Consequently, to create useful and usable data, there is a need to adopt measurement methods that are reliable, comparable and valid, for examining the relationship between patient safety culture and patient experience, such as the HSOPS and HCAHPS. It is also useful to consider qualitative investigation when exploring the relationship between these concepts.

### Relationship between patient safety culture and patient experience

Several relationships between patient experience and safety culture subdomains were identified in the included studies (Table 4). This suggests that staff and patient views on aspects of safety can be usefully incorporated and examined together. For example, the communication between staff and patients, and the coordination within and across hospital departments. According to Doyle, Lennox, and Bell [37], the smooth coordination (integration) of care is a key and valued aspect of the patient experience.

In this review, we found that the conceptual relationship between patient safety culture and the patient experience was not clearly described. The differences and overlaps between concepts, results, or measurement tools makes it difficult to understand the relationship between patient safety culture (among health professionals and managers) and patient experience. Future investigations may benefit from the development of a conceptual framework that allows researchers to test and develop their understandings of how patients’ experiences intersect with safety culture. We know that patient experience and safety culture are both valuable quality indicators. Better understanding how they are associated will enable healthcare staff to comprehend patient needs and create an effective strategy for enhancing patient safety culture that aligns with patients’ needs.

This scoping review has offered an overview of extant research regarding the association between patient experience and patient safety culture within the hospital context, and identified potential associations between the two concepts. However, the included studies have been conducted in limited countries, and generally assessed the relationship between these two concepts using quantitative methods. It may be the case that in other countries or cultures, the type of relationship could vary. Differences in ethnicity and national cultures could play an important role in patient experience. For instance, it was recognised in the reviewed literature, that Arab patients reported lower patient satisfaction levels compared with other ethnic groups within the same setting [10]. Therefore, it is important to consider other elements that may have an indirect effect on patient safety culture and patient experience, particularly in ethnic or national cultures where this relationship has not yet been investigated. Likewise, other factors related to the organisation could impact the relationship between the concepts. For example, the accreditation status of a facility has been shown to have a significant positive relationship with patient satisfaction [21].

## Conclusion

It has been demonstrated that the terms “safety culture” and “safety climate,” as well as “patient experience” and “patient satisfaction” are not always consistently applied across research, with the concepts not often being clearly defined, lacking a theoretical basis for the relationship, not being widely investigated with qualitative methodologies and with considerable diversity in terms of the tools and methodologies employed. The outcomes of this review suggest that research into the association between patient safety culture and patient experience needs to be investigated by using a suitable theoretical framework, in combination with validated methods, and supported by qualitative inquiry, in order to investigate this relationship more comprehensively, particularly in contexts where such investigations have not taken place.

### Limitations

While the literature search was conducted in major electronic databases without restrictions on date of publication or country of origin, additional relevant resources not in English or Arabic languages are likely to have been missed. This may lead to a language bias and limit the chance of capturing different perspectives from diverse communities to obtain a comprehensive understanding of the research phenomena, impacting the findings’ generalisability. Further, in accordance with the scoping review methodology of Arksey and O’Malley, a quality assessment was not conducted. Thus, it would be challenging to determine the validity of the reported findings due to the lack of quality assessment. These limitations are common in scoping reviews.

## Data Availability

Not applicable.

## Abbreviations

PRISMA-ScR: The Preferred Reporting Items for Systematic Reviews and Meta-Analyses Extension for Scoping Reviews
PCC: Population, Concept and Context
AHRQ: The Agency for Healthcare Research and Quality
ACSQHC: The Australian Commission for Safety and Quality in Healthcare
ACI: The Agency for Clinical Innovation
NIH: National Institutes of Health
HSOPS: The Hospital Survey on Patient Safety Culture
SAQ: The Safety Attitudes Questionnaire
PMOS: The Patient Measure of Safety
HCAHPS: The Hospital Consumer Assessment of Healthcare Providers and Systems
PSQ: The Patient Satisfaction Questionnaire Short Form
FFT: The Friends and Family Test
FS-ICU-24: Family Satisfaction in the Intensive Care Unit questionnaire
YCFF: The Yorkshire Contributory Factors Framework
